# Development of abbreviated measures to assess patient trust in a physician, a health insurer, and the medical profession

**DOI:** 10.1186/1472-6963-5-64

**Published:** 2005-10-03

**Authors:** Elizabeth Dugan, Felicia Trachtenberg, Mark A Hall

**Affiliations:** 1 The Division of Geriatric Medicine, the University of Massachusetts Medical School, Worcester, USA; 2 The Department of Public Health Sciences, Wake Forest University School of Medicine, Winston-Salem, USA; 3 The New England Research Institutes, Watertown, USA

## Abstract

**Background:**

Despite the recent proliferation in research on patient trust, it is seldom a primary outcome, and is often a peripheral area of interest. The length of our original scales to measure trust may limit their use because of the practical needs to minimize both respondent burden and research cost. The objective of this study was to develop three abbreviated scales to measure trust in: (1) a physician, (2) a health insurer, and (3) the medical profession.

**Methods:**

Data from two samples were used. The first was a telephone survey of English-speaking adults in the United States (N = 1117) and the second was a telephone survey of English-speaking adults residing in North Carolina who were members of a health maintenance organization (N = 1024). Data were analyzed to examine data completeness, scaling assumptions, internal consistency properties, and factor structure.

**Results:**

Abbreviated measures (5-items) were developed for each of the three scales. Cronbach's alpha was 0.87 for trust in a physician (test-retest reliability = 0.71), 0.84 for trust in a health insurer (test-retest reliability = 0.73), and 0.77 for trust in the medical profession.

**Conclusion:**

Assessment of data completeness, scale score dispersion characteristics, reliability and validity test results all provide evidence for the soundness of the abbreviated 5-item scales.

## Background

Trust is a key element of therapeutic relationships. Patient trust may influence health status through continuity of care, adherence to treatment regimens, the willingness to seek care [1,2], and perhaps via the mind~body pathway, which is not yet well understood. Biomedical researchers have paid increasing attention to trust as theoretical and measurement developments have occurred [3-45], driven in part by a concern about the potential negative influence of managed care on the doctor-patient relationship. Additional causes for concern about the doctor-patient relationship include the near daily release of conflicting health information about diet, lifestyle, or medications, and well-publicized, yet rarely occurring, outrageous examples of malpractice and medical errors.

The relationship between doctors and their patients has received philosophical, legal, and literary attention since Hippocrates, and is the subject of more than 8,000 articles monographs, chapters, and books in the modern medical literature [3]. At the conclusion of an extended, competitive, and expensive period of education and training required to enter the profession of medicine, physicians take a vow *to do no harm *to their patients. For more than a century the American Medical Association has had a code of ethics that states that the chief aim of medicine is to render service to humanity [26].

Mechanic has described trust as the "glue" that holds communities together and allows us to pursue our affairs without excessive suspicion, policing, and regulation [14]. We define patient trust as the optimistic acceptance of a vulnerable situation in which the patient believes the healthcare provider will take care of the patient's interests [5]. This recognizes that the patient-provider relationship involves vulnerability that stems from the experience of illness, the profound imbalance of knowledge and power, and the importance of what is at stake: one's health and well-being [24,25]. Put simply, if there is no vulnerability, there is no need for trust. The object of trust may be a healthcare provider, a hospital or clinic, a health insurance provider, or the medical system as a whole [5].

Our previous research reported on the development and validation of three instruments to measure trust in: 1) a doctor (or other healthcare provider) [6], 2) a health insurer [7], and 3) in doctors in general (e.g., the medical profession) [8]. Interested readers are directed to a detailed description of the conceptual framework of trust that guided this work [5]. Briefly, the framework posits that patient trust involves patients' vulnerability and their resulting reliance on and confidence in their physicians' competence, motivation, honesty, and confidentiality.

Despite the recent proliferation in research on patient trust, it is seldom a primary outcome, and is often just one of several peripheral areas of interest. Thus, the length of the original scales (10 and 11 items) limits the likelihood the scales will be widely used because of the frequent practical needs to minimize both respondent burden and research cost. Because of these concerns the present paper reports on the feasibility, factor structure, reliability and validity of abbreviated versions of the three instruments.

## Methods

### Samples

Sample 1: National sample. The first sample was selected by random-digit dialing. Inclusion criteria were: age ≥ 21; had health insurance (n = 151 excluded); had visited a healthcare provider at least twice in the past two years (n = 248 excluded); able to speak and understand English; and, able to complete a telephone survey. Contacts with the 2172 potentially eligible adults resulted in the following dispositions: 1117 (51%) provided verbal informed consent and were interviewed; 571 (26%) refused; 484 (22%) were unable to participate (e.g., no answer after 15 callback attempts, too ill, or not able to speak and understand English). Complete data were obtained from 1064 adults and were used in analyses.

Sample 2: North Carolina health maintenance organization (HMO) sample. The second dataset was a random sample of enrollees in a managed care plan who resided in North Carolina [9]. This sample included English-speaking adults aged 21 or older, who had been with the HMO for at least 2 years, and had made at least 2 visits to a primary care provider. Telephone contact was made with 1,908 (94.4%) resulting in the following dispositions: 319 (17%) were ineligible, 378 (20%) refused, and 1,211 (76%) provided verbal informed consent and agreed to participate. Complete data were obtained from 1,045 adults and were used in analyses. Two months later, a random subsample of 306 of these participants was resurveyed to assess test-retest reliability.

The telephone interviews averaged 35 minutes and were conducted by trained interviewers at the Survey Research Center of the University of South Carolina using computer assisted telephone interviewing. Verbal informed consent was obtained at the start of the telephone interviews. To ensure the adequate protection of human subjects in research, the study protocols were reviewed and approved by the Wake Forest University Medical Center Institutional Review Board.

### Measurement

The interviews collected information about patient and physician demographic characteristics; the name and type of health insurance; numerous items relating to trust in the subject's personal physician, including Kao's scale to measure physician trust in managed care members [20,21]; patient satisfaction with care [35]; single items to assess satisfaction in the doctor, insurer of interest, and doctors in general ("Overall, you are extremely satisfied with [doctor; health insurer; doctors in general]" coded (1) strongly agree to (5) strongly disagree). Also ascertained were self-reported adherence to doctor recommendations ("You always follow doctors' recommendations about treatment" responses: (1) strongly agree to (5) strongly disagree); whether one would recommend the doctor or insurer to family and friends (responses: (1) strongly agree to (5) strongly disagree); ever been upset or had a serious dispute with doctor [or, insurer] (yes, no); whether one had enough choice of doctor and insurer (yes, no); desire to switch doctor or insurer (yes, no); length of relationship with doctor and insurer of interest (number of years); self-reported physical and mental health (excellent, very good, good, fair or poor).

To reduce respondent burden the national sample was randomly divided; half were asked a battery of questions about health insurance trust, and the other half were asked a battery of questions about trust in doctors in general. Complete data were obtained from 410 adults on the health insurance trust items, and 502 adults on the medical system trust items. There were no statistically significant differences between the two samples on age, race, gender, or health status. All of the above measures were collected in the National sample, while the North Carolina sample did not collect information about trust in doctors in general, overall satisfaction, the willingness to recommend to family or friends, or the Kao trust scale.

### Statistical analyses

The abbreviated scales were developed using data from the national sample and then validated with data from both samples. The items were drawn from the original 10 or 11 item scales, which where constructed using psychometric analyses focused on feasibility, factor structure, validity, and reliability described in detail elsewhere [6-8]. This same approach was used to develop the abbreviated scales. Feasibility analyses examined data completeness, floor and ceiling effects, and the dispersion of scores. The item response distributions were examined. Items were deemed not feasible and dropped from the scale if there was a high rate of missing data or responses were concentrated in one or two categories indicating a lack of power to discriminate.

The objective was to develop a 5-item or shorter scale. Items were selected so that the abbreviated form: reflected the content of the conceptual model (competence, honesty, fidelity, and global trust); and contained both positively and negatively worded items. When two questions were otherwise equivalent, the one with the higher factor loading was chosen. Exploratory iterated principal components factor analysis with squared multiple correlations as initial communality estimates was performed to examine dimensionality. Items with absolute factor loadings of <0.60 were identified, and subsequent items were dropped until 100% of the variance was explained.

Correlations between the 5-item scale and the original 10 or 11 item scale were examined, as well as correlations between the 5-item scale and key theoretically determined concepts (e.g., Kao's trust scale, general satisfaction with care, number of years with doctor or insurer, with desire to switch doctor or insurer, number of visits, satisfaction with physician or insurer, willingness to recommend to friends, and whether doctors' recommendations are always followed). A two-sample t-test was used for those variables with a binary response format (e.g., prior dispute with doctor or health insurer, having changed doctors, any or enough choice in selecting doctor or health insurance, having sought a second opinion, and membership in managed care).

Internal consistency was determined by Cronbach's alpha. Test-retest reliability could only be calculated using data from the North Carolina HMO sample for the physician trust and health insurance trust 5-item scales.

## Results

A description of the two samples is reported in Table 1. The samples were similar in demographic characteristics; however the mean level of trust in insurer and physician in the North Carolina HMO sample was higher than that of the National sample.

**Table 1:** Demographic characteristics

	National sample (N = 1064)	HMO sample (N = 1045)
Age (mean)	49.75 years	46.56 years
Female	68%	55%
Hispanic ethnicity	5%	2%
Race		
African American	10%	12%
American Indian or Alaska Native	1%	1%
Asian or Pacific Islander	2%	.5%
White	84%	86%
Other	4%	0%
Education		
< High School	8%	6%
High School Graduate	28%	28%
>High School	64%	66%
Physical Health		
Excellent or Very Good	57%	58%
Good	28%	33%
Fair or Poor	14%	10%
Mental Health		
Excellent or Very Good	72%	72%
Good	24%	25%
Fair or Poor	5%	3%

*Percentages may not equal 100% due to rounding.

### Patient trust in a physician

#### Validity

Construct and concurrent validity were examined by correlation analyses and two-sample t-tests for items with binary responses. Table 2 reports the correlations for the 5-item scale in the National sample and the North Carolina HMO sample. Trust in a physician was correlated with: satisfaction with the physician; would recommend to friends and family; general satisfaction with care; no desire to switch to another doctor; number of years under physician's care; number of visits to physician. All correlations were significant at the p < 0.001 level. Binary validation analyses showed that trust was associated with having enough choice in the selection of the physician, not having had a dispute with the physician, and not having sought a second opinion due to concerns about care. Trust generally decreased with poorer physical health (Wilcoxon tests, p = 0.004). Trust also generally decreased with poorer mental health (Wilcoxon tests, p = 0.012). Trust did not vary by education level or income.

**Table 2:** The association of patient trust in a physician and key variables.

	**5-item scale national sample**	**5-item scale HMO sample**
Satisfaction with the physician	0.729	0.778
Would recommend physician	0.726	Na
General satisfaction with care	0.478	Na
Desire to change physicians	-0.660	-0.686
Number of years under dr.'s care	0.120	0.093
Number of visits to physician	0.127	0.150

Pearson correlation coefficients for continuous variables, Spearman correlation coefficients for categorical variables. All correlations significant at p < 0.001.

#### Reliability

The 5-item scale had a Cronbach's alpha of 0.87 in the National sample, and in the North Carolina HMO sample it was 0.86. As would be expected any time a scale is reduced in length, the reliability declined, albeit modestly. The 5-item scale had a lower internal consistency than either the original 10-item Wake Forest Scale (0.92) or the Kao scale (0.93).

#### Summary

There is strong evidence that a 5-item scale can be used to assess a patient's trust in her/his doctor. The 5-item scale is one-dimensional. Responses are summed and scores are on a 5–25 scale, with higher values indicating more trust. Ceiling and floor effects were acceptable. Flesch-Kincaid reading level of the scale was grade 4.3. The mean of the scale was 20.43, with a standard deviation of 3.13. The skewness was -1.05, and the reported kurtosis was 2.52.

### Trust in the medical profession

#### Validity

Construct and concurrent validity were examined by correlation analyses and two-sample t-tests (Table 3) . Trust in the medical profession was correlated with the Kao scale (r = 0.313), general satisfaction with care (r = 0.482), and following doctor's recommendations (r = 0.440).

The binary response validations showed that lower trust was related to having had a dispute with a physician, having changed doctors, and having sought a second opinion. Reported trust was lower for those with poorer mental health (Wilcoxon tests, p = 0.012). Trust did not vary by education level or income.

#### Reliability

Cronbach's alpha for the 5-item scale was 0.77. The 5-item scale has a lower internal consistency than the original 10-item scale, but is acceptable. No test-retest reliability data were available because the questions about trust in the medical profession were not included in the North Carolina HMO survey.

#### Summary

There is adequate evidence that the 5-item scale can be used to assess a patient's trust in the medical profession. The 5-item scale is one-dimensional. Responses are summed and scores are on a 5–25 scale, with higher values indicating more trust. Flesch-Kincaid reading grade level is 5.5. The mean of the 5-item scale was 14.97, with a standard deviation of 3.38. The skewness was -1.149, and the reported kurtosis was -0.330.

### Trust in a health insurer

#### Validity

Construct and concurrent validity were examined by correlation analyses and two-sample t-tests (Table 4) . Trust was correlated with Kao's trust scale (r = 0.279), general satisfaction with care (r = 0.465), satisfaction with health insurer (r = 0.646), and desire to find another health insurance provider (r = -0.753). Binary response validations showed that trust was related to having any choice in selecting health insurer, having enough choice in selecting health insurer, having a past dispute with the health insurer, and being in managed care. Adults with poorer mental health had significantly lower trust in their health insurance provider than adults in better mental health.

#### Reliability

The 5-item scale had a Cronbach's alpha of 0.84 in the National sample, and 0.83 in the North Carolina HMO sample. Test-retest reliability of the trust in health insurance provider 5-item scale was 0.729 in the general population.

#### Summary

There is evidence that the 5-item scale can be used to assess a patient's trust in a health insurer. Responses are summed and scores are on a 5–25 scale, with higher values indicating more trust. The mean score was 16.57 with a 3.94 standard deviation. The skewness was -0.729, kurtosis 0.339. Flesch-Kincaid reading grade level is 7.7. The scale is one-dimensional in the general population and explains 100% of the variance.

## Conclusion

Trust in a medical provider, a health insurer, and the medical profession may be influenced by many factors. As financing pressures continue to force the rapid evolution of the healthcare environment, particularly the patient-provider relationship, research to understand the consequences of such changes will only grow in importance. The 5-item scales developed in this study provide tools to facilitate such research.

Development of the 5-item scales was informed by our theoretical model and data driven. We sought to develop scales that provide sufficient measurement precision and breadth, yet minimize burden and cost. The scales are brief, comprehensive and empirically validated tools. The scales require reading levels of grades 4.3 (physician trust), 5.5 (medical profession trust), and 7.5 (health insurer trust). Each 5-item scale had acceptable psychometric properties.

Several limitations of the current research should be noted. First, the results reported here are for telephone administration of the scales. The performance of the scales in other settings is yet unknown. Second, the interviews were only conducted with English-speaking adults, although subsequent research is currently in press reporting on Spanish translations of some of these items. Further research on older adults, the most frequent users of healthcare, is urgently needed. Research to determine the effectiveness of interventions to enhance the doctor-patient relationship, and whether such enhancements, by extension, will improve important patient outcomes, is also needed.

## Appendix

### Patient trust in a physician

*1. Sometimes Dr._ [INSERT NAME OF DR.]__ cares more about what is convenient for (him/her) than about your medical needs.

2. Dr. _ [INSERT NAME OF DR.]_ is extremely thorough and careful.

3. You completely trust Dr._ [INSERT NAME OF DR.]'s decisions about which medical treatments are best for you.

4. Dr._ [INSERT NAME OF DR.]__ is totally honest in telling you about all of the different treatment options available for your condition.

5. All in all, you have complete trust in Dr._ [INSERT NAME OF DR.]_.

Response choices (coding) are: Strongly Agree (5), Agree (4), Neutral (3), Disagree (2), Strongly Disagree (1). Responses are summed (range 5–25) with higher scores indicating more trust. *Negatively worded item is reverse coded.

### Patient trust in the medical profession

*1. Sometimes doctors care more about what is convenient for them than about their patients' medical needs.

2. Doctors are extremely thorough and careful.

3. You completely trust doctors' decisions about which medical treatments are best.

4. A doctor would never mislead you about anything.

5. All in all, you trust doctors completely.

Response choices (coding) are: Strongly Agree (5), Agree (4), Neutral (3), Disagree (2), Strongly Disagree (1). Responses are summed (range 5–25) with higher scores indicating more trust. *Negatively worded item is reverse coded.

### Patient trust in a health insurer

*1. [INSERT NAME OF HEALTH INSURER] Cares more about saving money than about getting you the treatment you need.

*2. You feel like you need to double check everything [INSERT NAME OF HEALTH INSURER ] does.

3. You believe [INSERT NAME OF HEALTH INSURER] will pay for everything it is supposed to, even really expensive treatments.

4. If you have a question, you think [INSERT NAME OF HEALTH INSURER] will give you a straight answer.

5. All in all, you have complete trust in [INSERT INSURER'S NAME].

Response choices (coding) are: Strongly Agree (5), Agree (4), Neutral (3), Disagree (2), Strongly Disagree (1). Responses are summed (range 5–25) with higher scores indicating more trust. *Negatively worded item is reverse coded.

## Competing interests

The author(s) declare that they have no competing interests.

## Authors' contributions

Obtained research funding: MH, ED.

Research idea: ED.

Data collection: MH, ED

Statistical Analysis: FT.

Writing, revising, and final approval of manuscript: ED, MH, FT.

**Table 3:** The association of trust in the medical profession and key variables.

	**Original 11-item scale national sample**	**5-item scale national sample**
Kao's trust scale	0.306	0.313
General satisfaction	0.498	0.482
Follow doctor's recommendations	0.449	0.440
Original WFU 11 item scale		0.957

Pearson correlation coefficients for continuous variables, Spearman correlation coefficients for categorical variables. All correlations significant at p < 0.001.

**Table 4:** The association of trust in health insurer and key variables.

	**5-item scale national sample**	**5-item scale HMO sample**
Kao's trust scale	0.279	
General satisfaction	0.465	
Satisfaction with insurer	0.646	
Desire to switch insurers	-0.753	-0.589
Original WFU 10 item scale	0.952	0.948

Pearson correlation coefficients for continuous variables, Spearman correlation coefficients for dichotomous variables. All correlations significant at p < 0.001.

## Pre-publication history

The pre-publication history for this paper can be accessed here:


